# Atypical presentation of currarino syndrome: A case report

**DOI:** 10.1016/j.ijscr.2019.02.047

**Published:** 2019-03-18

**Authors:** Paul Hage, Cedric Kseib, Carmen Adem, Camil J. Chouairy, Reva Matta

**Affiliations:** a Department of Neurosurgery, Saint George Hospital University Medical Center, Balamand University, Beirut, Lebanon; b Department of Radiology, Saint George Hospital University Medical Center, Balamand University, Beirut, Lebanon; c Department of Pathology, Saint George Hospital University Medical Center, Balamand University, Beirut, Lebanon; d Department of Pediatric Surgery, Saint George Hospital University Medical Center, Balamand University, Beirut, Lebanon

**Keywords:** Malignancy, Dysraphism, Currarino syndrome, Neurosurgery, Pediatric surgery, Case report

## Abstract

**Introduction:**

Currarino syndrome is a rare congenital disorder characterized by a triad of anorectal malformation, a sacral bone defect, and a presacral mass. It results of an abnormal separation of the ectoderm from the endoderm caused by *HLXB9* mutation in chromosome 7q36 in 50% of cases. The disorder is mostly hereditary as it can also be sporadic with a variable expression spectrum.

**Presentation of Case:**

The case of a previously healthy 3-month-old girl with abdominal distension, post-prandial vomiting, obstipation, and anuria of 5 days’ history is presented in this article. Abdomino-pelvic magnetic resonance imaging (MRI) showed a large cystic multilobulated mass in the sacrococcygeal region with a dural communication evident of an anterior sacral meningocele. 1 year later, the child came back with constipation and was found to a have a malignant mixed germ cell tumor in the presacral area, a very rare presentation in Currarino syndrome.

**Discussion:**

In a child presenting with at least one of the features of Currarino syndrome’s triad, a diagnosis should be suspected. After reviewing the literature, the syndrome is usually missed and hence is under diagnosed. MRI is the best imaging modality for diagnostics and follow-up for any mass, benign or malignant, can bring life saving measures. Most masses are benign but can undergo malignant transformation even after resection. De novo malignancy is very rare and is described in our case.

**Conclusion:**

Physicians treating patients with spinal dysraphism should suspect a diagnosis of Currarino syndrome by follow up imaging for any new benign or malignant growth.

## Highlights

• Currarino syndrome is a rare congenital disorder characterized by a triad.
• MRI is the best imaging modality in early diagnosis and follow up for recurrences.
• The presacral mass can be a malignancy in Currarino syndrome.
• Both neurosurgery and pediatric surgery are needed in tackling Currarino syndrome.

## 1 Introduction

Currarino syndrome is a rare congenital disorder characterized by a triad of anorectal malformation, a sacral bone defect, and a presacral mass. It is the result of an abnormal separation of the ectoderm from the endoderm caused by *HLXB9* mutation in chromosome 7q36 in 50% of cases [[1], [2], [3]]. The disorder is mostly hereditary as it can also be sporadic with a variable expression spectrum. We report a case of a previously healthy 3-month-old girl who presented with abdominal distension, post-prandial vomiting, obstipation, and anuria of 5 days’ history. Abdomino-pelvic magnetic resonance imaging (MRI) showed a large cystic multilobulated mass in the sacrococcygeal region with a dural communication evident of an anterior sacral meningocele. 1 year later, the child came back with constipation and was found to a have a presacral malignant mass. This work was reported in line with the SCARE criteria [4].

## 2 Case report

**Fig. 1:**
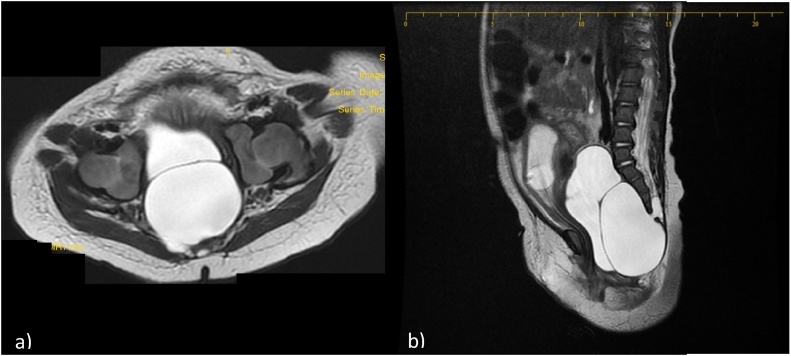
1a and 1b: Axial and sagittal T2-weighted images showing a septated cystic lesion located within the pelvis. This lesion is in continuity with the spinal canal through a hiatus located in the anterior and right aspect of the sacrum. (Horizontal line on the sagittal view). Dysraphism of the sacrum and coccyx is noted. Note that the urinary bladder contains a Foley catheter and is significantly compressed, as well as anteriorly and superiorly displaced.

A previously healthy 3-month-old girl was transferred to our hospital for severe abdominal distention, post-prandial vomiting, obstipation, and anuria for the last 5 days. CT scan done prior to presentation at another hospital showed a cystic abdominal mass displacing the girl’s bowels, bladder globus, and bilateral hydroureteronephrosis (not shown). Her creatinine level was elevated reaching 4.99 mg/dl upon admission. An abdomino-pelvic MRI showed an 8.2*3.7*3.2 cm homogenous cystic multilobulated pelvic mass in the sacrococcygeal area with a 1.5*0.4*0.6 cm dural canal communicating with the mass at the S4-S5 level consistent with an anterior sacral meningocele (Fig. 1). To restore kidney function and prevent renal failure, a urinary foley was inserted and was successful in dropping her creatinine levels to normal reaching 0.28 mg/dl the third day.

Surgical resection was performed on the fifth day with a posterior approach starting with an incision from S3 to the coccyx and a laminectomy to expose the sacral canal. The dural communication was ligated from the rest of the thecal sac followed by cyst cerebrospinal fluid drainage.

The next day, post-operation echography showed residual cysts in the intra-abdominal cavity. Abdominal laparoscopy was done on the eleventh day to drain the remaining cysts which enabled urinary foley removal and patient’s discharge symptoms free.

**Fig. 2:**
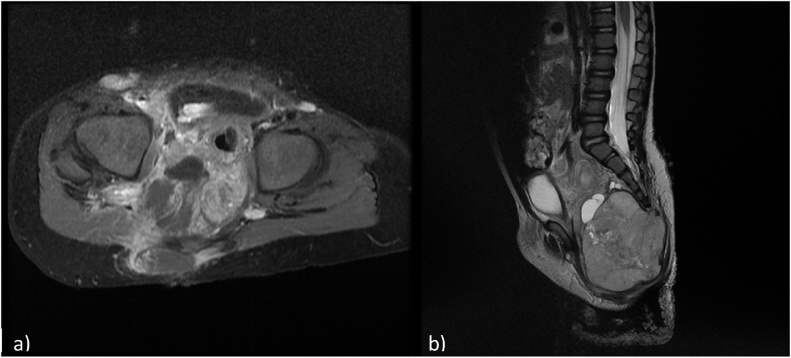
2A and 2B: Axial T1 weighted image with fat signal suppression following gadolinium administration showing a partially enhancing, large lesion located in the pelvis, in the pre-and post sacral spaces. Sagittal T2 weighted image showing a mixed large lesion with a dominant solid component located at the tip of the sacrum showing an extension to the pelvis and posterior subcutaneous tissues.The urinary bladder is again noted to be anteriorly and superiorly displaced.

**Fig. 3:**
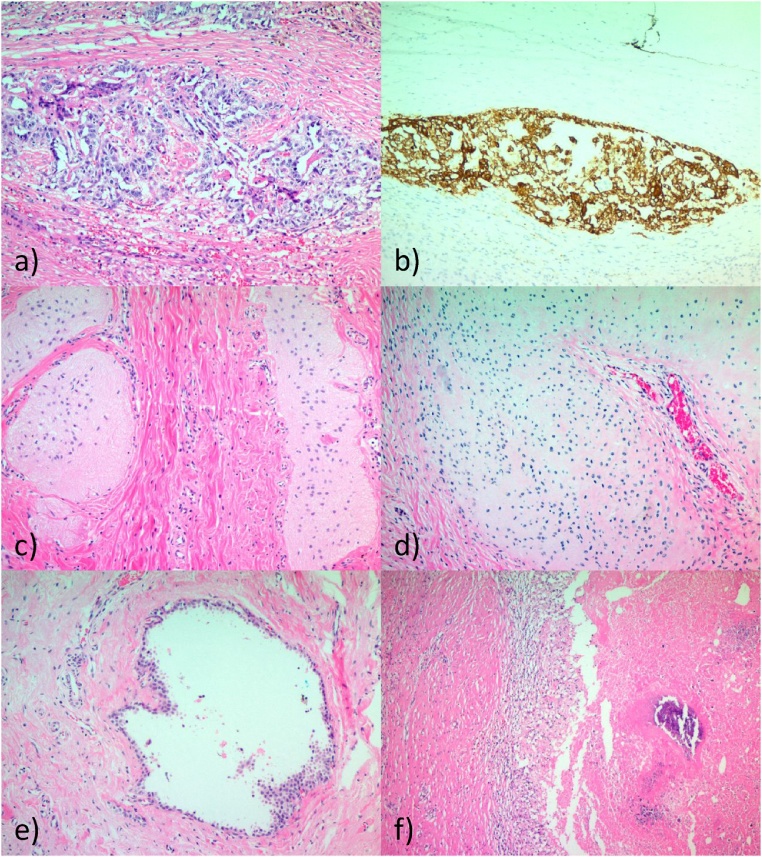
Pathology of tumor. Residual focus of viable yolk sac tumor exhibiting solid growth pattern with glandular formations (a), anti-CK positive and CD30 negative (b), residual glial tissue (c), residual mature cartilage (d), cystically dilated gland lined by simple benign cuboidal to columnar epithelium at 100x magnification (e) with necrosis and dystrophic calcification and histiocytes at 50x magnification with H&E stain (f).

A year later, the child presented back with constipation. A lumbosacral MRI showed a solid lesion of 7.5 cm of height and 6.5 cm of diameter associated with adenopathies (Fig. 2). An inguinal lymph node biopsy demonstrated the presence of a yolk sac tumor. Neoadjuvant chemotherapy was started. 3 months later the tumor was resected. On pathology, the tumor was found to be an extragonadal germ cell tumor composed of mature benign glial tissue, endodermal derived tissue bone and cartilage with admixed yolk sac tumor, diagnostic of malignant mixed germ cell tumor (Fig. 3).

## 3 Discussion

Currarino syndrome is an autosomal dominant congenital malformation defined by a triad described by Currarino et al. in 1981 consisting of a sacral bony defect, a presacral mass, and an anorectal malformation or constipation [5] with only around 300 cases reported [6,7]. It results from an incomplete separation of the endodermal and ectodermal layers during embryo development causing a connection between the gut and the spinal column due to failure of anterior vertebral fusion [1]. The phenotypic presentation is variable as it can be incomplete with 1 or 2 anomalies of the triad making the diagnosis easily missed. The disorder can also be sporadic. Mutations of the *HLXB9* homeobox gene on chromosome 7 (7p36) was found to be involved in 90% of familial Currarino [6] and in around 30% of sporadic cases [8]. Our patient is a sporadic case since she is the first in her family to have the disorder.

The presacral mass can be anterior sacral meningocele (ASM), teratoma, and/or neuro-enteric, dermoid, and epidermoid cysts. ASM occurs as a result of a sacrococcygeal defect or herniation of the meningeal sac from the sacral foramen to the anterior and occasionally with neural elements into the pelvis presenting as a presacral pelvic mass [1]. Our patient first presented with an ASM with a sacrococcygeal defect.

Although rarely symptomatic, local pressure by the ASM may cause constipation, obstipation, and urinary retention. Second, local neurological deficits may present as poor sphincter control, sacral anesthesia, or lower extremity deficit. Finally, central neurologic symptoms may present as nausea and headache with straining due to increased abdominal pressure on the meningocele. Our patient presented with severe abdominal distention, acute urinary retention, obstipation, and post-prandial vomiting, due to the increased pressure in the abdomen from the ASM that was detected by MRI evaluation [2]. Neurosurgical approach is recommended for symptomatic lesions as in our patient, especially if there are neurological deficits, bowel and bladder involvement, and/or an increase in the size of the lesion due to increased hydrostatic pressure. A sacral laminectomy, anterior abdominal approach, or laparoscopic treatment by anterior or posterior approach may be used with the cooperation of pediatric and neurological surgeons for optimal result [9]. The ASM connection was obliterated by a posterior approach by the neurosurgeon for better dural closure and decreased risk of contamination of the spinal fluid should the bowel be injured. Due to the multiloculated nature of the ASM, a laparoscopic anterior approach was performed to drain the remaining cysts by the pediatric surgeon to fully decompress the bowel and bladder.

Part of the Currarino triad is anorectal malformation causing constipation or obstipation which pathogenesis is attributed to either mechanical compression by a presacral mass, a tethered cord, a sacral nerve roots compression, or an anteriorly located anus [1,8,10]. The rapid resumption of both obstipation and anuria after ASM compression removal explained the mechanical obstruction of both bowel and bladder.

MRI is best imaging modality in detecting the communication between the ASM and the spinal subarachnoid space due to high soft tissue contrast and multiplanar imaging. It also helps exclude any other pathologies associated with ASM such as a presacral mass [11]. Bony defects are best visualized with CT-scan. Ultrasonography can be used in only diagnosing ASM since it does not give detailed characteristics of the lesion nor able to visualize the communication.

Malignancy is very rare in Currarino syndrome and may involve malignant transformation of a benign mass with only 10 cases being reported, 6 of them during childhood [6,12]. The rate of malignant transformation in Currarino syndrome is estimated at around 1% of documented cases [13]. Children with malignant transformation were under 5 years at diagnosis after 1 year of excision of an apparent benign tumor [6]. Only one case of malignant teratoma was cited in the literature as a first presentation [7]. Of the malignancies reported we found malignant teratoma, leiomyosarcoma, and neuroendocrine tumor [13]. In our case, after a year of the ASM resection, the presacral mass was found to be a malignant mixed germ cell tumor, first case to be reported within the Currarino syndrome. No masses were detected during the ASM resection making it also the first case of de novo appearance of a malignant tumor. Due to the infrequency and missed recognition of Currarino syndrome, recurrence of a tumor should be kept as a possibility. Follow-up with MRI imaging is recommended in Currarino to detect any appearance of a pelvic mass.

## Conflict of interest

Nothing to declare.

## Funding

Balamand University will only pay for publishing fees for the journal if manuscript is accepted; otherwise no funding was involved in the writing and conduction of the article.

## Ethical approval

The study is exempt from ethical approval by our institution.

## Consent

Written informed parental consent was obtained on behalf of the patient since patient is a minor for publication of this case report and accompanying images. A copy of the written consent is available for review by the Editor-in-Chief of this journal.

## Author contribution

- Dr. Paul Hage and Dr. Cedric Kseib: study concept, data collection, data analysis, writing of the manuscript.
- Dr. Reva Matta: data collection and interpretation.
- Dr. Carmen Adem: imaging studies provision and interpretation.
- Dr. Camil J. Chouairy: pathology images and interpretation.

## Registration of research studies

Nothing to declare.

## Guarantor

Dr. Paul Hage, Neurosurgeon.

## Provenance and peer review

Not commissioned, externally peer reviewed.
